# Invasion dynamics of the European bumblebee *Bombus terrestris* in the southern part of South America

**DOI:** 10.1038/s41598-021-94898-8

**Published:** 2021-07-27

**Authors:** Francisco E. Fontúrbel, Maureen M. Murúa, Lorena Vieli

**Affiliations:** 1 Instituto de Biología, Pontificia Universidad Católica de Valparaíso, Valparaiso, Chile; 2 Centro GEMA: Genómica, Ecología y Medio Ambiente, Facultad de Estudios Interdisciplinarios, Universidad Mayor, Santiago, Chile; 3 Departamento de Ciencias Agronómicas y Recursos Naturales, Butamallin Research Center for Global Change, Universidad de La Frontera, Temuco, Chile; 4 Center of Applied Ecology and Sustainability (CAPES), Santiago, Chile

**Keywords:** Community ecology, Invasive species

## Abstract

Invasive species are one of the main biodiversity loss drivers. Some species can establish and thrive in novel habitats, impacting local communities, as is the case of managed pollinators. In this regard, an invasive species' expansion process over time is critical for its control and management. A good example is the European bumblebee *Bombus terrestris*, which has rapidly invaded the southern part of South America after being repeatedly introduced in Chile for crop pollination since 1997. We assessed the temporal dynamics of *B. terrestris* invasion in Argentina and Chile by compiling 562 occurrence points from 2000 to 2019. We used two estimators (minimum convex polygon and 95% fixed kernel) to estimate the increase of the invaded area over time. We found that the area invaded by *B. terrestris* in the southern part of South America presents a linear increase over time, which was consistent for both estimators. In this scenario, species traits, environmental characteristics, and introduction dynamics facilitate a rapid invasion process that will continue to expand, reaching other South American countries in the near future. As this bumblebee is a super-generalist, it probably will expand across South America, as climate niche modelling predicts, if no actions were taken.

## Introduction

Biological invasions are acknowledged as one of the main biodiversity loss drivers^1^. While many exotic species are moved outside their native distribution, only a few of them successfully establish, depending on the affinity with the target habitat^2,3^. However, those species that successfully invade a given habitat usually spread fast after surpassing a population density threshold, becoming difficult to control beyond that point^4^. For that reason, timing is crucial in invasive species management as there may be a point of no return^5^. The invasion process depends on the habitat invasion susceptibility (i.e., environmental characteristics), the species invasibility (certain traits make some species more prone to invade), and introduction dynamics (repeated introduction events results in high propagule pressure)^6–8^. Besides, many exotic species can perform better than native species in novel environments due to their generalism and plastic responses; those species are more prone to invade successfully, easily invading disturbed habitats that present less biotic resistance^9^. Moreover, the arrival of an invasive pollinator species is likely to alter the topology of native plant-pollinator networks^10^. For example, it has been observed that generalist pollinators such as *Bombus terrestris* (Linnaeus, 1758) and *Apis mellifera* (Linnaeus, 1758) can be easily integrated into native pollination networks^11–14^. In fact, the easier a given species is integrated into this network, the more probable it is to invade and establish new mutualist interactions over time.

**Figure 1:**
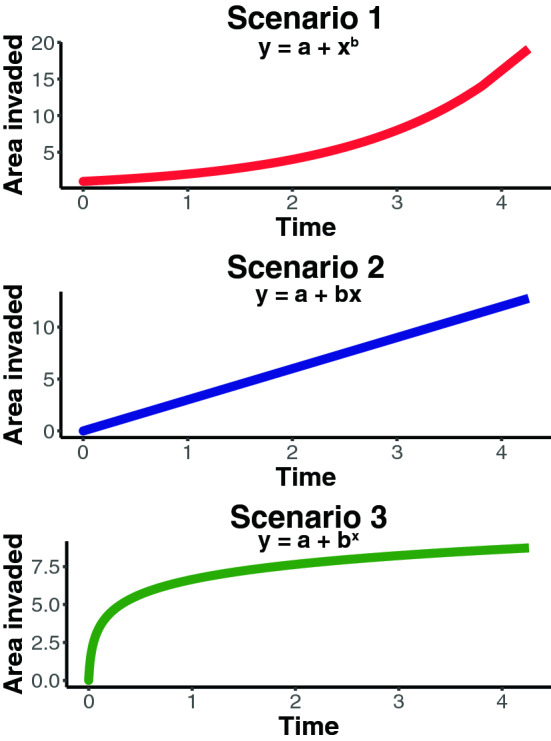
Theoretical scenarios of invasive species spread, based on exponential, linear, and power generic functions.

When we consider the geographic expansion of an invasive species over time, we may find different invasion patterns depending on the invasive species' life-history traits, the characteristics of the novel environment (i.e., habitat suitability), and the number and location of introduction sites^7^. However, thinking on a more general level, a species undergoes three distinct stages during its invasive dynamics regardless of each species' specific traits or responses: an initial establishment phase, an expansion phase, and a saturation phase^15^. This pattern is often repeated over time at different spatial scales^16^, explaining lags and episodic expansions of most invasive species. These stages reflects different spatial expansion dynamics that can be represented with three simple theoretical scenarios (Fig. 1). The first scenario shows an exponential increase of the area invaded, in which the establishment of new populations is initially slow, and the expansion accelerates over non-invaded areas. The second scenario shows a constant increase in the area invaded, with a linear expansion of the area occupied across time. The third scenario shows an initial increase of the area invaded, slowing down until it reaches a plateau, following a power function. Each phase's temporal persistence and velocity depend on species traits, environmental conditions, biotic interactions, and stochasticity^17,18^. Also, the introduction dynamics (e.g., number and location of introduction events) are relevant to determine the spread of an invasive species^7^.

A well-known case of species invasion is the buff-tailed bumblebee *B. terrestris*, a eusocial insect native to Eurasia and northern Africa. It is one of the most common and abundant bumblebee species in the northern hemisphere. This species has been intentionally introduced, and still is, to several countries for crop pollination purposes and has successfully established and invaded parts of Japan, Mexico, and China, among others^19^. This bumblebee is acknowledged as an efficient pollinator for different plant species^20,21^, and for this reason, it is massively bred to be used to supplement pollination in greenhouses and crop fields around the world^19^. *Bombus terrestris* was introduced in Chile in 1997 to pollinate tomato crops^22^. Initially, this exotic pollinator was aimed for greenhouse pollination only. However, in a short time, its use in open-air crops was authorized by the Chilean Agriculture and Livestock Bureau (SAG, by its acronym in Spanish), becoming invasive and spreading throughout the country in a few years. Since 1997, more than 320.000 colonies and one of million queens have been introduced to Chile (information obtained from the Chilean Agriculture and Livestock Bureau, under the Public information transparency act, request AR006T0005510 issued by LV in January 2021); however, the information about the exact locations and dates of those introductions is not available, as the local authorities do not require farmers to deliver such information until 2020 (since 2021 the new regulation made mandatory to supply this information). Therefore, the constant introduction of new *B. terrestris* colonies for crop pollination in south-central Chile subsidize the existing naturalized populations, possibly reinforcing its genetic diversity. While Argentina banned *B. terrestris* introduction, it became rapidly invaded by the spillover from Chile^23^. For those reasons, *B. terrestris* provides a suitable study model to assess the invasion dynamics at a continental scale due to its rapid expansion in the southern part of South America and its potential to invade other countries across the continent (Brazil and Uruguay in the short term, but reaching until Colombia and Panamá in the long term), based on climatic niche modelling^24^.

In this study, we assessed the current spatial dynamics of *B. terrestris* expansion, aiming to determine the current invasion phase for this species. Considering the rapid expansion of *B. terrestris* across the southern part of South America in the last decade and that this particular case meets the three invasibility conditions described by Pyšek et al.^7^ (i.e., a generalist species with certain traits, a suitable environment, and an introduction dynamics with constant introductions), we hypothesized that its invasion dynamics would resemble the first scenario, with an exponential increase in the area invaded over time.

## Results and discussion

**Figure 2:**
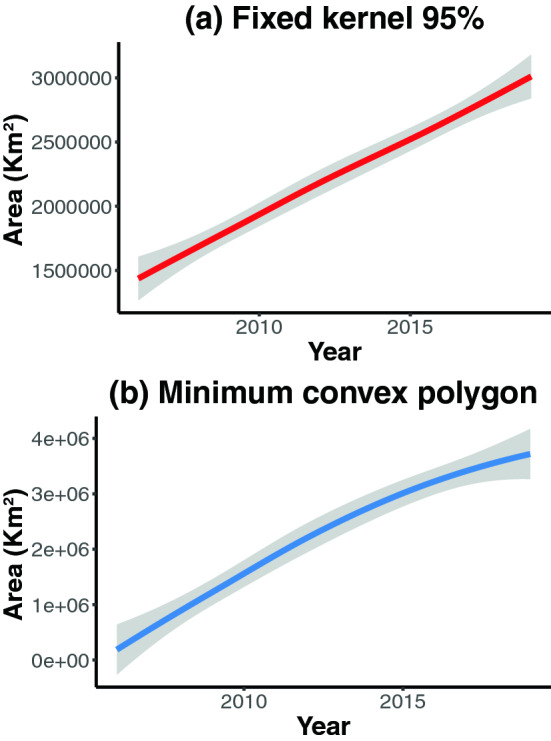
*Bombus terrestris* occupied area over time (smoothed values).

**Figure 3:**
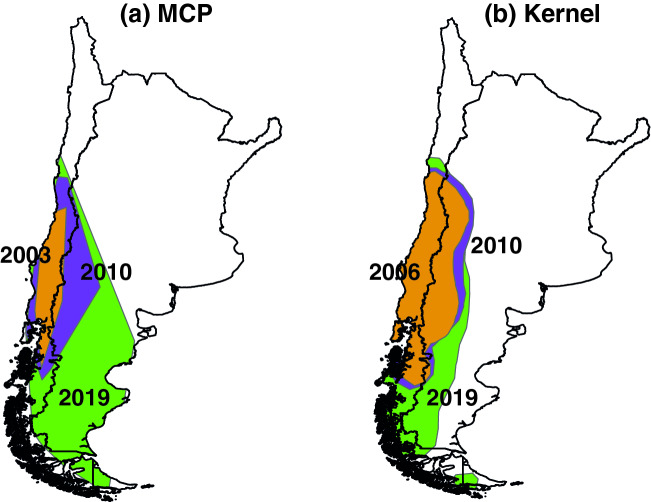
*Bombus terrestris* area in three different times, using both minimum convex polygon (MCP) and 95% fixed kernel (FK) estimators. (this map was created by FEF in QGIS 3.18 [www.qgis.org], using our own data).

We obtained a total of 562 *B. terrestris* occurrence points for Chile and Argentina (Table S1 and Fig. S1, available online as Electronic Supplementary Material). The number of occurrence points has also increased over time between 2003 and 2019 (Fig. S2). Examining the areas occupied by *B. terrestris* over time, we see an increment according to both minimum convex polygon (MCP hereafter) and 95% fixed kernel (FK hereafter) estimators (Figs. 2 and Fig. S3). The increase in the invaded area is more evident using the MCP estimator than the FK estimator (Fig. 3). Further, we found that the number of points and the area are strongly correlated for both MCP (r = 0.987, *P* < 0.001) and 95% fixed kernel (r = 0.977, *P* < 0.001) estimators.

Our MCP and 95% FK results show that *B. terrestris* invasion resembles the second scenario (fit to a linear function: MCP adjusted R^2^ = 0.94; FK adjusted R^2^ = 0.96), as the invaded area had a constant increase over time. Besides, examining raw area data (Fig. S3), we observe some periods of rapid expansion, which is coherent with the stratified diffusion process described by Leibhold et al.^16^, in which the three stages of invasion (i.e., arrival, establishment, and expansion) are continuously repeating across spatial (from local to continental) scales. In any case, *B. terrestris* in the southern part of South America is still expanding its range, rapidly increasing across different habitats (i.e., sclerophyllous scrublands, dry forests, temperate rainforests, and the Patagonian steppe) in Argentina and Chile^23,25,26^. This super-generalist bumblebee can invade a wide range of habitats, taking advantage of a wide variety of flower resources^27,28^.

Despite being different estimators^29^, MCP and 95% FK results are similar and indicate that *B. terrestris* expansion over the southern part of South America increased over time, and it is likely to continue increasing in the forthcoming years. A similar *B. terrestris* expansion trend was found in Japan^30^ (which is the only study addressing this issue before ours). Considering the predictions made by Acosta et al.^24^ using a niche-modeling approach, it is likely that *B. terrestris* will continue expanding its range beyond Chile and Argentina, reaching other South American countries, being Brazil and Uruguay the most affected in the short term.

Information quality is one of the main limitations of this kind of analysis. Probably the records obtained are biased as the different research groups and citizen observations tend to oversample some locations while many others remain undersampled. This issue can be resolved (at least in part) by relying on other information sources such as citizen science^31^. As *B. terrestris* is a conspicuous species, easy to identify by non-expert volunteers, citizen science programs may provide valuable information to track *B. terrestris* spread, making occurrence data more representative by reducing sampling bias inherent to scientific monitoring due to time and resource limitations.

The negative impacts of this invasive species have been extensively documented, both in Chile and worldwide^19^. Previous studies in Chile revealed that its invasion could drastically disrupt some native plant-pollinator relationships with important consequences to plant fitness. For example, in the Andean Maule mountain, it has been observed that *B. terrestris* is capable of breaking the reproductive barriers between two specialized *Calceolaria* plant species inhabiting in sympatry, favouring the formation of sterile hybrids^32^. Similarly, *B. terrestris* has been documented as a primary nectar robber of the native sub-scrub *Fuchsia magellanica,* decreasing the visitation of native pollinators and negatively affecting their reproductive success^33^.

The worrisome scenario shown by our results might be largely underestimated as there is no formal tracking of this invasive bumblebee, and the available records are mostly opportunistic. We urge the authorities to establish an invasive species monitoring program to properly track the expansion of *B. terrestris* populations already established in these countries (along with other invasive species). Also, it is imperative to stop importing new colonies since the arrival of new individuals strengthens their genetic diversity propitiating the appearance of new genotypes able to cope unfavourable environmental conditions and successfully colonize new habitats. It is also important to regulate *B. terrestris* beekeeping practices in Chile, keep records of their use, and make such information available for monitoring purposes. In a global change framework, land-use change and climate change may have a synergic effect on biological invasions, facilitating this kind of insect's invasion process. Furthermore, *B. terrestris* and other large bumblebees are expected to become dominant species with climate warming^34^, worsening the future invasion scenario.

By providing evidence of the current expansion phase of *B. terrestris* in Chile and Argentina, it is possible to forecast that the ecological impacts of introducing this species in Chile—continuously since 1997—will have even more intense detrimental ecological effects, which will impact beyond the current distribution of this species. The continuous importation of new *B. terrestris* colonies to supplement pollination services for Chilean agriculture represents an input of new individuals, increasing genetic and pathogen diversity, worsening the current situation. Such agricultural practices, which prioritize the use of exotic pollinators instead of the native ones, are subject to little and weak governmental regulation, has favoured a subsidiary invasion dynamic that caused a major invasion problem in Argentina and are likely to affect other South American countries (e.g., Bolivia, Perú, Uruguay, and Brazil) in the near future. The constant supplementation of exotic bumblebees should the stopped as soon as possible, as ecological damage will continue to increase if no action is taken^22^. Therefore, international environmental regulations should focus on the multilateral consequences of this kind of invasion process and seek effective and coordinated regulation^23^.

## Methods

### Data collection

We searched the literature for articles reporting *B. terrestris* occurrences, providing coordinates and year of the record. We searched the Web of Science and Scholar Google databases, using the search terms "Bombus terrestris" + Chile and "Bombus terrestris" + Argentina. After filtering non-relevant results or articles providing incomplete information (e.g., no coordinates or no dates), we selected 18 articles^25,26,33,35–49^ that provided 201 occurrence records. Besides, we obtained 172 records from a citizen science project (Salvemos Nuestro Abejorro, https://salvemosnuestroabejorro.wordpress.com/), kindly provided by José Montalva as the raw data were not available in the original article^50^. We also searched Global Biodiversity Information Facility (GBIF) for *B. terrestris* occurrences, which provided 189 additional records with year and coordinates. We created derived dataset on April 5th 2020, registered under https://doi.org/10.15468/dl.f7jezh for this purpose^51^. The complete database is available in Table S2.

The *B. terrestris* invasion problem in Chile and the subsequent invasion to Argentina began with intentional and repeated introductions for crop pollination^23^. However, the information regarding the dates and locations of those introductions is not available as the national authorities do not require farmers to deliver that information. While some of the records obtained may be associated with those crops, most of the records correspond to non-crop areas (Fig. S1) and can be interpreted as a natural spread. The same reasoning applies to the invaded area in Argentina, as this country has prohibited the importation of *B. terrestris* colonies.

### Data analysis

With the occurrence data gathered, we built a matrix with the record's year and the geographic coordinates (standardized in decimal degrees). Then, we separated records per year; we excluded those from 1998 to 2003 due to the low number of occurrences in this period. Therefore, we kept records from 2006 to 2019 only. Then, we created point shapefiles with the occurrences per year. Those shapefiles are incremental (i.e., the occurrence points of a given year include those from the past years). We used two approaches to estimate the area occupied by *B. terrestris* each year: the minimum convex polygon (MCP, using 100% of the locations) and a fixed kernel (FK, using 95% of the locations). Both approaches have advantages and disadvantages^29^, and we decided to use both of them and compare their results. We used the adehabitatHR package in R 3.6.3^52^ to calculate MCP and FK areas. We trimmed areas to the continent to avoid overestimating areas when resulting polygons presented portions covering the ocean. We assessed the correlation between the number of occurrences and the area estimated using the Pearson correlation coefficient. We used QGIS (version 3.18, obtained from www.qgis.org) to generate the maps with our own shapefiles.

## Availability of data and code

Original data and shapefiles generated are available from the *figshare* digital repository https://doi.org/10.6084/m9.figshare.12959825.

## Supplementary Information

Supplementary Information.
