# Supplementary material for: Invasion dynamics of the European bumblebee Bombus terrestris in the southern part of South America
Source: Sci Rep. 2021 Jul 27;11:15306. doi: 10.1038/s41598-021-94898-8 (PMC8316498; doi:10.1038/s41598-021-94898-8)
Supplement: Supplementary file 1 — Supplementary Information. [file 41598_2021_94898_MOESM1_ESM.pdf]

ELECTRONIC SUPPLEMENTARY MATERIAL

Invasion dynamics of the European bumblebee *Bombus terrestris* in the southern part of South America

Francisco E. Fontúrbel, Maureen M. Murúa & Lorena Vieli

Table S1. Cumulative number of occurrences per year.

Year	Occurrence points
2006	22
2007	36
2008	67
2009	108
2010	152
2011	246
2012	319
2013	379
2014	389
2015	401
2016	423
2017	436
2018	477
2019	525

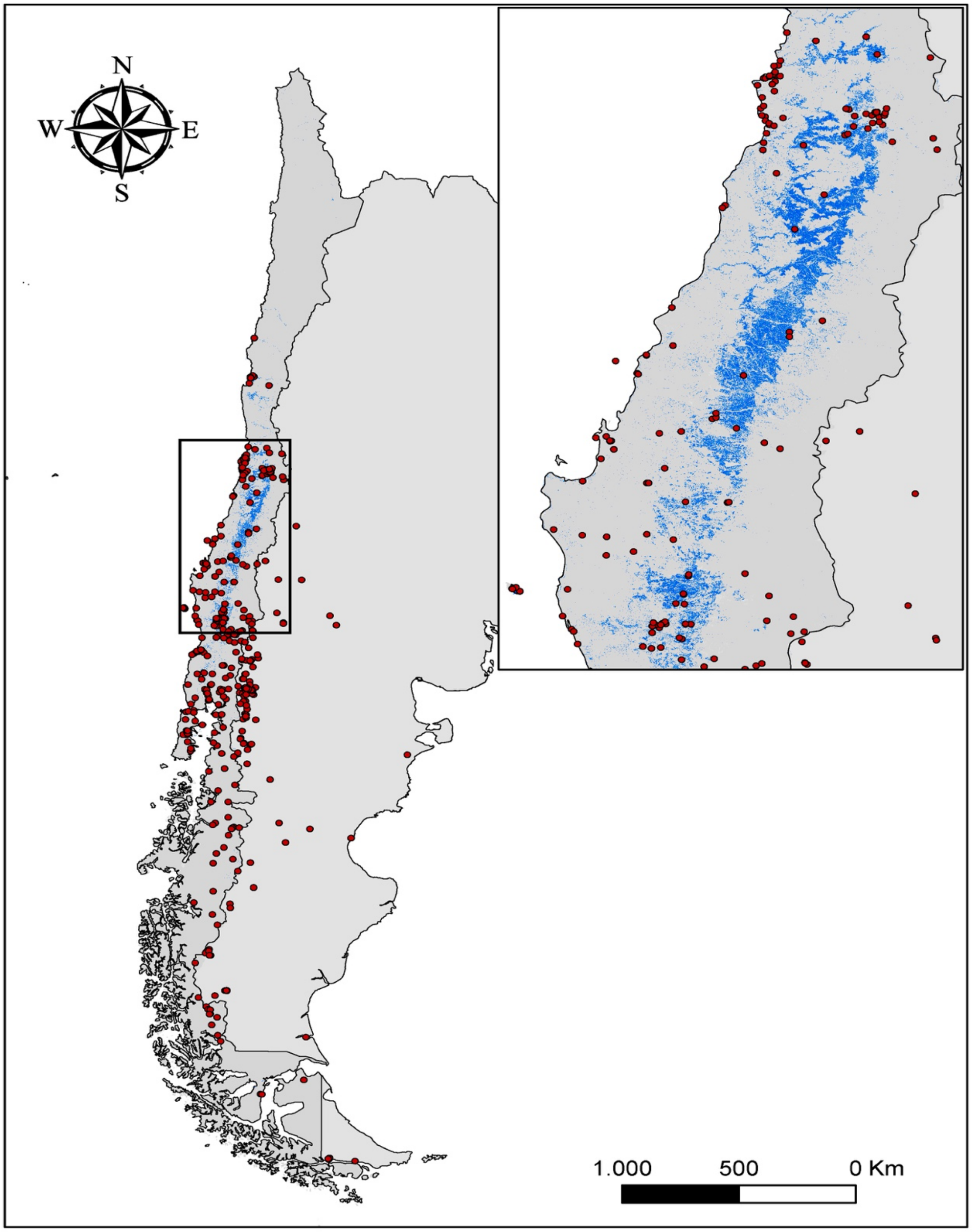

Fig. S1. *Bombus terrestris* occurrence (red dots) in the southern part of South America. The area depicted in blue correspond to pollinator-dependent crops in Chile, and the inset focuses on central Chile, where most agriculture is concentrated (this map was created by LV in QGIS 3.18 [www.qgis.org], using our own data).

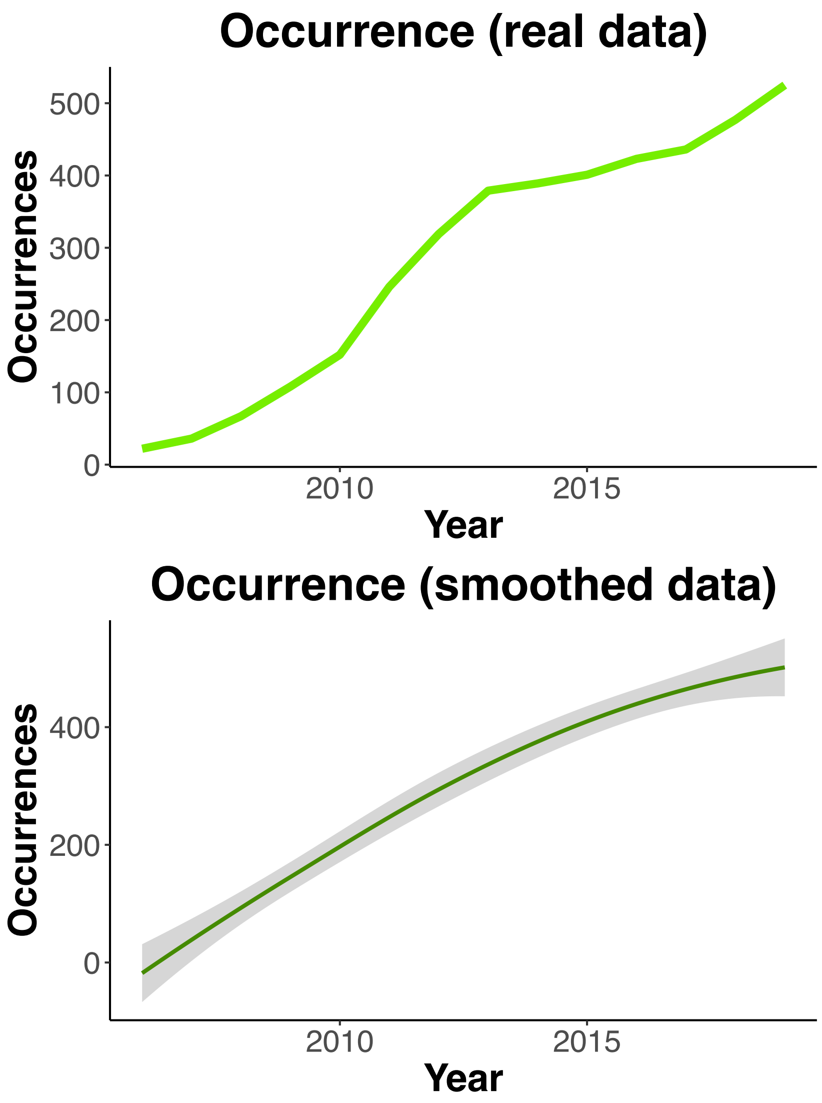

Fig. S2. *Bombus terrestris* occurrence change over time.

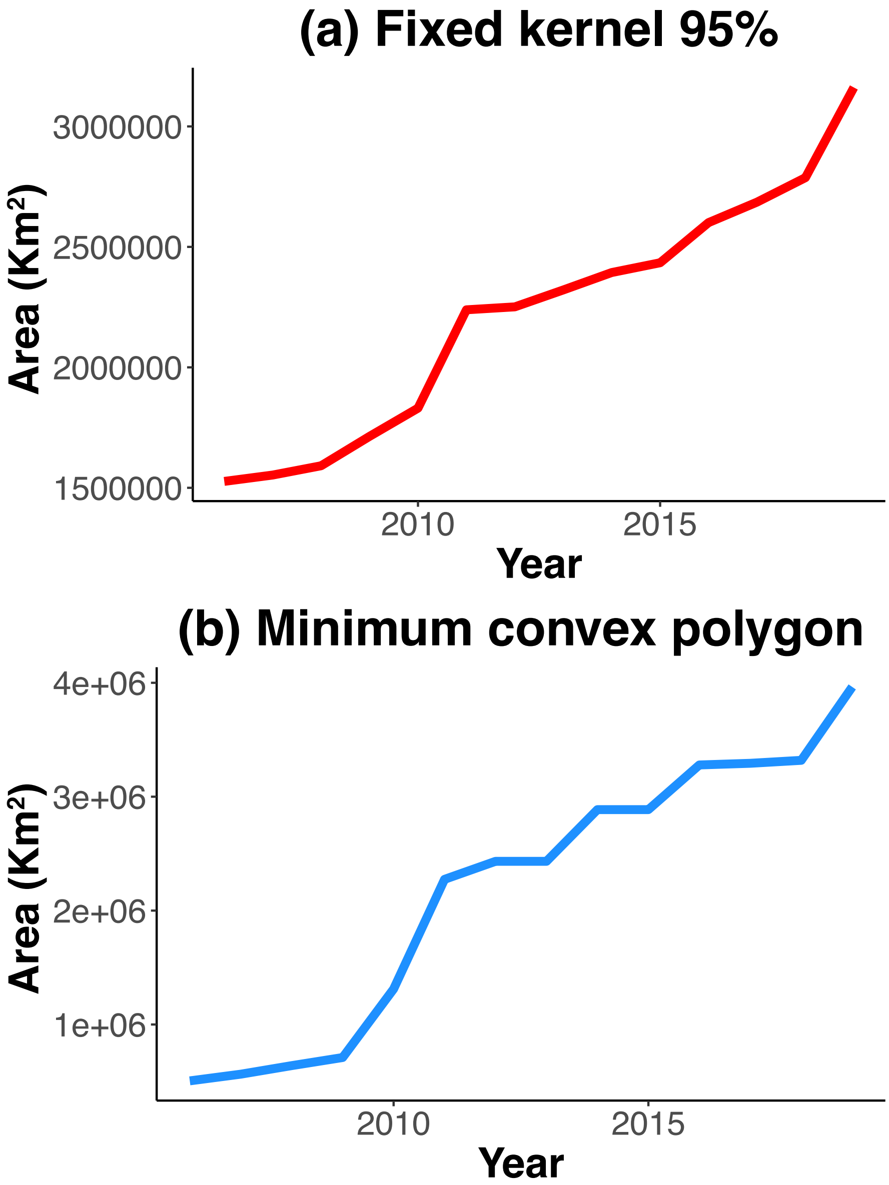

Fig. S3. *Bombus terrestris* occupied area over time (raw values).

Table S2. Data sources from which we obtained 562 *Bombus terrestris* occurrence records.

Source	Date range	Occurrence points
Ruz & Herrera (2000)	2000	4
Torreta et al. (2006)	2006	1
Plischuk et al. (2009)	2009	1
Plischuk et al. (2011)	2009-2010	2
Esterio et al. (2013)	2010-2012	2
Morales et al. (2013)	2011	29
Aizen et al. (2014)	2012	3
Sáez et al. (2014)	2012	1
Schmid-Hempel et al. (2014)	2010-2012	28
Geslin & Morales (2015)	2011-2014	7
Polidori & Nieves (2015)	2013	1
Vieli et al. (2015)	2011	9
Arismendi et al. (2016)	2014-2015	4
Valdivia et al. (2016)	2012-2013	108
Montalva et al. (2017)	2011	165
Plischuk et al. (2017)	2009-2016	5
Sáez et al. (2017)	2014-2015	2
Sáez et al. (2018)	2013	1
GBIF dataset	1998-2020	189

Data source citations:

Ruz L & Herrera R (2000) Preliminary observations on foraging activities of Bombus dahlbomii and Bombus terrestris (Hymenoptera: Apidae) on native and non-native vegetation in Chile. Acta Horticulturae 561: 165-169

Torretta JP, Medan D & Abrahamovich AH (2006) First record of the invasive bumblebee Bombus terrestris (L.) (Hymenoptera, Apidae) in Argentina. Transactions of the American Entomological Society 132(3): 285-289

Plischuk S & Lange CE (2009) Invasive Bombus terrestris (Hymenoptera: Apidae) parasitized by a flagellate (Euglenozoa: Kinetoplastea) and a neogregarine (Apicomplexa: Neogregarinorida). Journal of Invertebrate Pathology 102(3): 263-265

Plischuk S, Meeus I, Smagghe G, Lange CE (2011) Apicystis bombi (Apicomplexa: Neogregarinorida) parasitizing Apis mellifera and Bombus terrestris (Hymenoptera: Apidae) in Argentina. Environmental Microbiology Reports 3(5): 565-568

Esterio G, Cares-Suárez R, González-Browne C, Salinas P, Carvallo G & Medel R (2013) Assessing the impact of the invasive buff-tailed bumblebee (Bombus terrestris) on the pollination of the native Chilean herb Mimulus luteus. Arthropod- Plant Interactions 7: 467–474

Morales CL, Arbetman MP, Cameron SA, Aizen MA (2013) Rapid ecological replacement of a native bumble bee by invasive species. Frontiers in Ecology and the Environment 11(10): 529-534

Aizen MA, Morales CL, Vázquez DP, Garibaldi LA, Sáez A, Harder LD (2014) When mutualism goes bad: density-dependent impacts of introduced bees on plant reproduction. New Phytologist 204(2): 322-328

Sáez, A., Morales, C. L., Ramos, L. Y., & Aizen, M. A. (2014) Extremely frequent bee visits increase pollen deposition but reduce drupelet set in raspberry. Journal of Applied Ecology 51(6): 1603-1612

Schmid-Hempel R, Eckhardt M, Goulson D, Heinzmann D, Lange C, Plischuk S, ... Schmid-Hempel P (2014) The invasion of southern South America by imported bumblebees and associated parasites. Journal of Animal Ecology 83(4): 823-837

Geslin B, Morales CL (2015) New records reveal rapid geographic expansion of Bombus terrestris Linnaeus, 1758 (Hymenoptera: Apidae), an invasive species in Argentina. Check List: 11, a1620

Polidori C, Nieves-Aldrey JL (2015) Comparative flight morphology in queens of invasive and native Patagonian bumblebees (Hymenoptera: Bombus). Comptes Rendus Biologies: 338(2), 126-133

Vieli L, Davis FW, Kendall BE, Altieri M (2016) Landscape effects on wild Bombus terrestris (Hymenoptera: Apidae) queens visiting highbush blueberry fields in south-central Chile. Apidologie: 47(5), 711-716

Arismendi N, Bruna A, Zapata N, Vargas M (2016) Molecular detection of the tracheal mite Locustacarus buchneri in native and non-native bumble bees in Chile. Insectes Sociaux 63(4): 629-633

Valdivia CE, Carroza JP, Orellana JI (2016) Geographic distribution and trait- mediated causes of nectar robbing by the European bumblebee Bombus terrestris on the Patagonian shrub Fuchsia magellanica. Flora: 225: 30-36

Montalva J, Sepulveda V, Vivallo F, Silva DP (2017) New records of an invasive bumble bee in northern Chile: expansion of its range or new introduction events?. Journal of Insect Conservation 21(4): 657-666

Plischuk S, Antúnez K, Haramboure M, Minardi GM, Lange CE (2017) Long-term prevalence of the protists Crithidia bombi and Apicystis bombi and detection of the microsporidium Nosema bombi in invasive bumble bees. Environmental Microbiology Reports 9(2): 169-173

Sáez A, Morales CL, Garibaldi LA, Aizen MA (2017) Invasive bumble bees reduce nectar availability for honey bees by robbing raspberry flower buds. Basic and Applied Ecology 19: 26-35

Sáez A, Morales JM, Morales CL, Harder LD, Aizen MA (2018) The costs and benefits of pollinator dependence: empirically based simulations predict raspberry fruit quality. Ecological Applications 28(5): 1215-1222

GBIF derived dataset (April 15, 2020) https://doi.org/10.15468/dl.f7jezh
